# Neuroantibody Biomarkers: Links and Challenges in Environmental Neurodegeneration and Autoimmunity

**DOI:** 10.1155/2014/340875

**Published:** 2014-06-23

**Authors:** Hassan A. N. El-Fawal

**Affiliations:** Neurotoxicology Laboratory, Albany College of Pharmacy and Health Sciences, 106 New Scotland Avenue, Albany, NY 12208, USA

## Abstract

The majority of neurodegenerative (ND) and autoimmune diseases (AID) remain idiopathic. The contribution of environmental chemicals to the development of these disorders has become of great interest in recent years. A convergence of mechanism between of ND and AID development has also emerged. In the case of ND, including neurotoxicity, the focus of this review, work over the last two decade in the realm of biomarker development, indicates that the immune response provides a venue whereby humoral immunity, in the form of autoantibodies to nervous system specific proteins, or neuroantibodies (NAb), may provide, once validated, a sensitive high throughput surrogate biomarker of effect with the potential of predicting outcome in absence of overt neurotoxicity/neurodegeneration. In addition, NAb may prove to be a contributor to the progression of the nervous system pathology, as well as biomarker of stage and therapeutic efficacy. There is a compelling need for biomarkers of effect in light of the introduction of new chemicals, such as nanoengineered material, where potential neurotoxicity remains to be defined. Furthermore, the convergence of mechanisms associated with ND and AID draws attention to the neglected arena of angiogenesis in defining the link between environment, ND, and AID.

## 1. Introduction

Identification of etiological factors that precipitate autoimmune (AID) and neurodegenerative diseases (ND) continues to be a challenge. According to the World Health Organization (WHO), 1 in 6 individuals, worldwide, suffer from a neurological disorder, mostly idiopathic, while the prevalence of AID varies according to the organ/system affected. WHO has prioritized investigations of the link between the environmental factors (e.g., chemicals) and both these disease entities [1, 2]. This is paralleled by the recently formulated strategic plan of National Institute of Environmental Health Sciences (NIEHS) in the study of environmental links to both ND and AID [3, 4]. The debilitating impact, as well as social and economic burden, particularly in children and the elderly, is compelling reason to develop biomarkers that can translate to the clinical setting in order to diagnose, evaluate sequelae, and provide a means of measuring successful intervention and to the identification of etiological factors and defining the mechanisms involved in ND and AID. It has become evident in recent years that there is a convergence of mechanisms involved in the pathogenesis of many ND and AID. Central to both is the increased angiogenesis and autoinflammatory sequelae to tissue damage. Also relevant to both is the involvement of integrins and Th17 lymphocytes. Additionally, the involvement of oxidative stress and necrotic-apoptotic events with exposure of autoantigens and the ensuing inflammation strengthens the proposition that the immune system may be a major effector of neurodegeneration. What is acknowledged in ND and AID is the loss and/or alterations in structural proteins, organ/cell-specific or common antigens, and an autoimmune, often humoral, signature. Indeed, because many of these proteins are sequestered intracellular proteins, the presence of immune effectors at the site of injury results in a humoral immune response (i.e., immunoglobulins (Ig)) directed against these autoantigens has been demonstrated in response to environmental chemical exposures. Work in our laboratory over the course of two decades has demonstrated that autoantibodies to NS proteins (neuroantibodies) may provide biomarkers of injury and may possibly be pathogenic [5].

It should be noted that in the context of this review and neurotoxicity, the discussion focuses on the effects of known environmental and occupational chemicals known to directly cause nervous system damage and not the recently described autoimmune/inflammatory syndrome induced by adjuvants (ASIA). Yehuda Shoenfeld's [6] group coined the term ASIA, also known as Shoenfeld's syndrome, as an umbrella to describe the clinical conditions of siliconosis, Gulf War syndrome, macrophagic myofasciitis syndrome, sick building syndrome, and postvaccination phenomena which share similar signs or symptoms, some of which are neurological and may be associated with demyelination and the presence of autoantibodies to an adjuvant material. The premise in ASIA is that the adjuvant may set in motion biological and immunological events that, in susceptible individuals, ultimately lead to the development of autoimmune disease, whereas in neurotoxicity we are often dealing with chemicals that directly induce neuronal death, apoptotic and necrotic, glial dysfunction, and aberrant neurotransmission [5].

This review seeks to address a major challenge in the identification and diagnosis of neurotoxicity and, by extension, ND, which is the development and validation of biomarkers of nervous system insult. However, in so much that these suggested biomarkers rely on an immune response to autoantigen (i.e., an autoimmune response), a possible epiphenomenon secondary to insults, whether acute or chronic, it raises the question as to whether this response may prove to be pathogenic, contributing to progression of the neuropathology. It should be noted that the study of neurotoxicity provides a useful paradigm for the development and testing of potential biomarkers of nervous system insult, since the level of injury can be controlled by dose, in the case of preclinical models, and the etiological factor(s) with target selectivity (neuronal versus demyelination) can be defined based on the agents used.

## 2. The Generation of Neuroantibodies as Biomarkers

**Figure 1:**
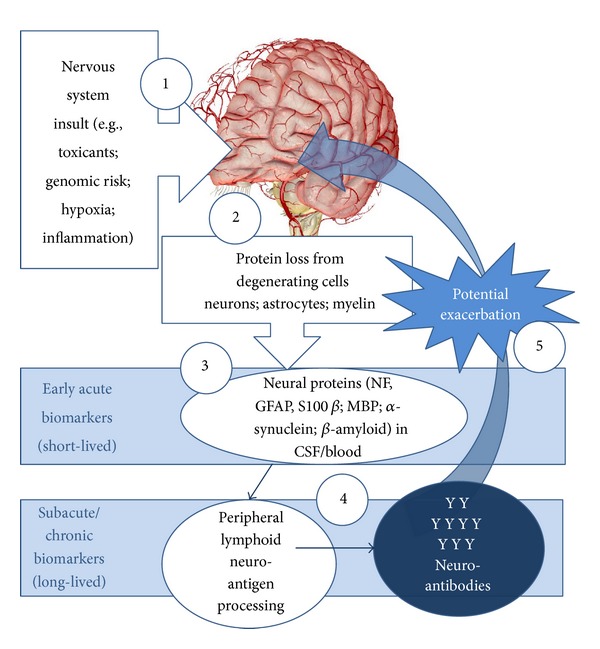
(1, 2) In the presence of toxicant-induced neurodegeneration, alterations in intracellular structural proteins and proteolysis, antigens (e.g., neuronal neurofilaments, *α*-synuclein, *β*-amyloid) are released into (3) the CSF and blood where (4) they are processed by antigen-presenting cells (APC) in the lymphoid tissue to induce humoral autoimmune responses. This autoimmune response, manifested in the form of autoantibodies, provides an accessible biomarker of neurotoxic effects [5]. These antibodies may propagate neurodegenerative changes through complement activation and direct targeting of the neural and vascular architecture, particularly in the presence of increased vascular permeability. Modified from [5].

Since proteins, many of them intracellular, are invariably lost during the neurodegenerative process and/or neurotoxic insult, several studies have advocated for the detection of proteins in cerebrospinal fluid (CSF) and blood serum or plasma as biomarkers of neurodegeneration. This has been reviewed elsewhere [7, 8]. This approach assumes that the clinician is aware of the precipitating event (e.g., stroke, traumatic brain injury, toxic exposure). However, the reality is that functional deficits are often slow in development and indeed, in the case of Alzheimer's disease (AD), Parkinson's disease (PD), and amyotrophic lateral sclerosis (ALS), may take years before a diagnosis is made based on overt morphological and behavioral alterations, when intervention may be of limited benefit. Unfortunately, this approach has its limitations, most notably the short half-life of many of these proposed proteins in the periphery and/or their specificity for NS as shown in Table 1 [9*-*13]. In contrast, because many of these proteins are sequestered intracellular proteins, the presence of immune effectors* in situ* or following translocation to peripheral lymphoid tissue (i.e., cervical lymph nodes and spleen) results in a humoral immune response directed against these autoantigens. With the development of immunological memory (IgG) or chronic degeneration (IgM), these immunoglobulins are likely to persist. Thus, capitalizing on the immune response, work in the Neurotoxicology Laboratory has advocated and demonstrated that these autoantibodies (neuroantibodies or NAb) provide a stable signature of nervous system (NS) injury [5]. This hypothesis is summarized in Figure 1.

However, once again, the question may be raised, if NAb are validated as biomarkers of effect, what advantage do they provide for the clinician and when would they be applied? In the context of exposure to known neurotoxicants, particularly occupational exposures, exposure monitoring, individual and environmental, is common as required by the Occupational Safety and Health Administration (OSHA) in the United States and the European Agency for Safety and Health at Work (EU-OSHA). This requires periodic monitoring of workers and their rotation within the industry based on exposure levels (e.g., blood Pb). It is conceivable that a validated high-throughput biomarker of effect may be a useful adjunct to routine exposure monitoring in occupational settings. This is particularly relevant with the increased evidence linking environment and ND, as well as gene-environmental interactions. In addition, many countries now require the determination of blood Pb levels in school aged children. Environmental and occupational chemicals, solvents, polychlorinated biphenyls (PCB), methyl mercury, lead, and pesticides are known to be neurotoxic in adults and developmental neurotoxicants in children, yet diagnosis of effects is often delayed until overt behavioral manifestations occur, despite knowledge of exposure histories. The availability of a validated biomarkers of insult would prove beneficial to detect early effects in vulnerable populations. Today, monitoring of blood cholesterol, hemoglobin A1C (HbA1c), and prostate-specific antigen (PSA), once validated, have become routine tests to evaluate risk of cardiovascular disease, diabetes, and prostate cancer, respectively. It is conceivable that a validated biomarker of nervous system insult, such as NAb, may come to enjoy such a status. Indeed, the need for routine, economical, relatively noninvasive blood-based biomarkers, with early testing, in absence of overt signs, has been advocated in the case of AD, as a result of a roundtable convened in 2012 by the Alzheimer's Association and the Alzheimer's Drug Discovery Foundation [14]. In this context, it should be noted that the threshold (i.e., quantity of autoantigen) to produce an immune response is significantly less than the magnitude of neuronal loss to detect overt clinical manifestations (e.g., 60–80% nigrostriatal 3 dopaminergic neuronal loss in PD, [15]).

The parallels between neurodegeneration and neurotoxicities, including the generation of NAb, have recently been reviewed [5] and will not be detailed here. This has included preclinical and clinical studies of acute and chronic exposures to heavy metals (inorganic lead, methyl mercury, trimethyl tin) organic compounds (insecticides, solvents, and organophosphorus compounds) and in hemodialysis patients [16–24]. Rather, this review will use examples to highlight the utility of NAb and future challenges in the sphere of neurotoxicity, ND, and AID.

## 3. Approach

For the toxicologist, experimental and clinical, pursuing the development and use of biomarkers, it is of great benefit to bear in mind two guiding principles. The first of these is the association between the biomarker and the associated cellular substrate in terms of relevance. For the neurotoxicologist, even if evaluating an environmental exposure of unknown neurotoxic potential, proteins unique to the NS should be identified. While many investigations in the last two decades have measured blood cytokine levels with interest in a particular system insult, because of investigator specialty, the relevance of these cytokines can only be revealing if tied into that system of interest by a measure unique to that system. It also becomes incumbent on the investigator that she/he recognizes that, for environmental exposures, multiple organs may be targeted by toxic compounds. For examples, PCB or lead may target the liver, the kidney, reproductive system(s), and immune and nervous systems. The detection of biomarkers against the liver, the kidney, or reproductive system(s) does not minimize or preclude the relevance and toxicity of having detected biomarkers that indicate NS involvement, only because other organ systems are involved.

The second principle to bear in mind, particularly with the increased interest in gene-environment, environment-ND, and environment-AID associations, is that preclinical and prospective clinical toxicology studies provide a model paradigm to develop and validate emerging biomarkers for genetic/genomic risk, ND, and AID. While strides have been made in developing biomarkers of ND, if one looks at the AD, PD, or ALS literature, these biomarkers are, more often than not, developed in patients with overt ND, robbing them of their predictive value as to disease outcome. However, with a “training set” of environmental chemicals, if a biomarker is developed that indicates the likelihood of neurodegenerative changes* prior* to emergence of frank deficits and pathology, such a biomarker would likely prove of benefit in defining idiopathic ND, as well.

A useful approach in the development and validation of NAb has been predicated on three tiers.

1. Do NAb indicate neuropathology, regardless of a specific etiological factor (toxicity, physical trauma, ND)? This recognizes that there are common protein substrates (autoantigens) found in all neurons (e.g., neurofilaments (NF)), astrocytes (e.g., GFAP), and elaborated by myelinating cells (e.g., myelin basic protein (MBP)).
2. Can NAb that identifies a unique cellular target (e.g., cholinergic versus catecholaminergic neurons)? This recognizes that there are neurotransmitter proteins, particularly enzymes (e.g., acetylcholinesterase (AChE), tyrosine hydroxylase), neuron-specific structural proteins (e.g., DARP-32 in dopaminergic neuron), or protein aggregates (*α*-synuclein, *β*-amyloid). It also recognizes that different neurotoxicants and neurodegenerative conditions target different populations of neural cells.
3. Are NAb pathogenic? This recognizes that autoantibodies which may be epiphenomena, secondary to injury, have frequently been shown to have agonistic or antagonistic activity, bind (and penetrate) cells, as well as activate complement and other immune effectors.

Whenever possible, it is prudent to look for concordance between experimental and clinical studies. The study and figures below demonstrate the tiered approach using organophosphate-induced delayed polyneuropathy (OPIDP) as an example.

## 4. Application 

In this proof of concept approach, the Neurotoxicology Laboratory has chosen the detection of NAb against proteins representing the cellular heterogeneity of the nervous system, while being common to all neurons, regardless of specialization based on neurotransmitters. Some of these protein antigens and the cellular substrates they represent are summarized in Table 2.

Organophosphorus compounds represent a large class of chemical agents that include insecticides and nerve agents, as well as chemicals that are used as lubricants, fuel, and industrial additives. The acute toxicity of the insecticides and nerve agents due to severe AChE and pseudoesterase inhibition has long been recognized. Chronic low level exposure may induce what is known as an intermediate syndrome, whereas acute single exposures of lubricant, fuel, and industrial additives may induce a central-peripheral neuropathy known as OPIDP. This is believed to be independent of anti-AChE activity, although some AChE inhibitors may also precipitate OPIDP. We have previously published result of NAb utility in OPIDP induced by phenyl saligenin phosphate (PSP) and amelioration with calcium channel blockade in the hen, the Environmental Protection Agency's (EPA) mandated model [23]. These results have been confirmed in humans by AbouDonia and colleagues [25].

**Figure 2:**
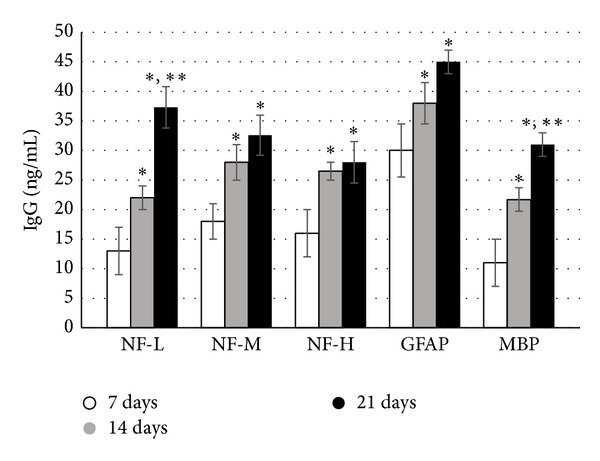
Serum titers of IgG against neurotypic (NF) and gliotypic (GFAP and MBP) proteins in hens (*n* = 7) administered a single dose of OPIDP-inducing phenyl saligenin phosphate (2.5 mg/kg, im). Serum was collected at 7, 14, and 21 days. Mean levels of IgG (±S.E.) were significantly (^∗^ *P* < 0.05) higher at 14 and 21 days compared to 7 days. With the exception of anti-NF-L and anti-MBP titers, there were no statistical differences between 14 and 21 days, suggesting peaking of the anti-NF-M, anti-NF-H, and anti-GFAP titers against these antigens. There was no detectable titer of antibodies against these antigens in control hens or in sera of hens prior to PSP administration.

**Figure 3:**
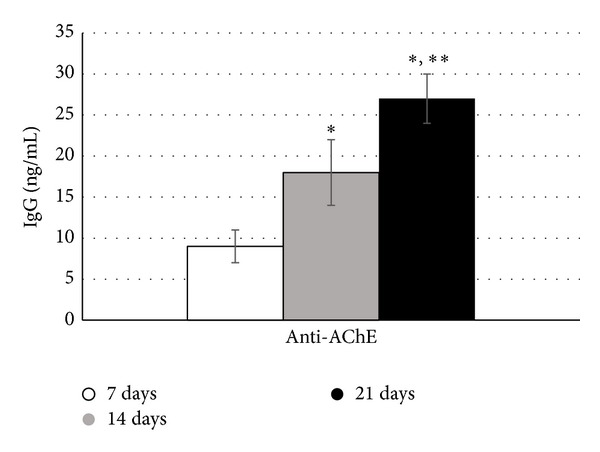
Serum titers of IgG against AChE, the enzyme responsible for acetylcholine hydrolysis, in hens (*n* = 7) administered a single dose of OPIDP-inducing phenyl saligenin phosphate (PSP, 2.5 mg/kg, im). Serum was collected at 7, 14, and 21 days. Mean levels of IgG (±S.E.) were significantly (^∗^ *P* < 0.05) higher at 14 and 21 days compared to 7 days and at 21 days compared to 14 days (^∗∗^ *P* < 0.05). Although OPIDP development is believed to be independent of AChE inhibition, some organophosphates may induce acute inhibition and phosphorylation of the enzyme.

**Figure 4:**
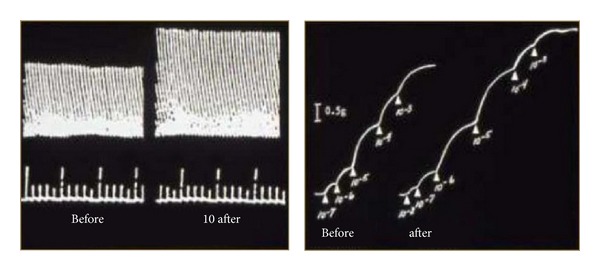
Representative original polygraph tracings of chicken biventer cervicis nerve-muscle preparation (in Krebs-Henseleit solution aerated with 95% O_2_/5% CO_2_) in response to electrical stimulation of fast twitch fibers and contraction to exogenous acetylcholine (slow muscle fibers) in the presence (after) and absence (before) of pooled immunoglobulin fraction (21 days after PSP administration). This suggests that these serum immunoglobulins may alter neuromuscular function.

**Figure 5:**
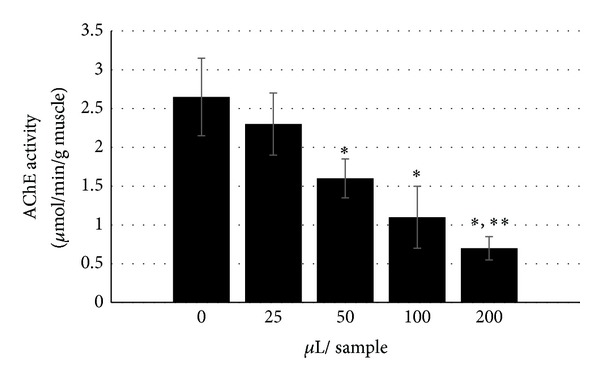
Activity of skeletal muscle homogenate AChE in the presence of different volumes of pooled serum Ig fraction from hens (*n* = 7) at 21 days following a single exposure to OPIDP-inducing phenyl saligenin phosphate (PSP, 2.5 mg/kg, im). This confirms that serum elements, possibly the detected anti-AChE, may interfere with neuromuscular function as observed in the isolated muscle preparations (see Figure 4).

In a study confirming our 2008 [23] findings, NAb, IgG (avian IgY), against NF, GFAP, and MBP were detected in hens as early as 7 days following a single dose of PSP (Figure 2). Titers of anti-NF, but not GFAP or MBP, NAb significantly correlated with changes in gait considered indicative of ataxia in OPIDP, decrease in stride length, and increase in width (Table 3). The lack of associations with GFAP and MBP is consistent with the primary targeting of neurons in OPIDP, although the earlier study did show associations with anti-MBP, secondary myelin involvement, and scored clinical ataxia [23, 26]. In addition, IgG against AChE was detected in sera of these hens (Figure 3). In a preliminary study, Ig fraction separated by dialysis, when incubated with the biventer cervicis nerve-muscle preparation [26] increased the magnitude of twitch responses of fast muscle fibers to electrical stimulation, as well as the response of slow muscle fibers to exogenous acetylcholine (Figure 4). This indicates that serum elements may have had inhibitory activity on catabolic enzyme or agonistic activity. This was further confirmed, in part, by measuring muscle homogenate AChE activity in the absence and presence of dialysis fractions of pooled sera with detectable anti-AChE antibodies (Figure 5).

It is important to insert a word of caution regarding the use of autoantibody detection in the context of toxicological studies, in general, and for neurotoxicology, in particular. Classical toxicology (and pharmacology) is quite often wed to dose, where an increase in dose beyond threshold is predicted to result in an increase in response and by extension deficits. While, in our experience, this is frequently the case, it quite often may not hold true when measuring an immune response. Several factors are likely to account for this. In brief, the following should be recognized.

1. Traditional measurements of internal dose, exposure biomarkers, to an environmental agent often reflect cross-sectional measurements (single time point), ignoring the kinetics and accumulation of the agent and where it may be sequestered (e.g., blood lead). They do not necessarily reflect past exposures or biological response. Borrowing from the virology and vaccine literature [27], in developing and validating NAb as biomarkers of neurotoxicity, the question is that given a particular exposure are these autoantibodies present and do they exceed background or the “natural repertoire” of antibodies. In the latter case, determination of antibody class and subclass is also likely to be useful.
2. The immune response, cellular and humoral, is a dynamic processes, but not infinite. Classical studies of immunoglobulin responses to vaccines recognize fluctuations and peaking of immunoglobulin production to a given antigen. It is likely that autoantibody levels peak even in the presence of continued exposure or accumulation of toxic chemicals.
3. Antibodies changes in affinity and avidity with repeated exposure to antigen and the development of immunological memory. It is useful in preclinical studies to harvest lymphoid tissue for cell isolation and* in vitro* challenge. It should also be recognized that antigens have multiple epitopes which impacts on polyclonal titer levels, as well as the exposure of new epitopes that may not be exposed in the native protein(s).
4. In the presence of high levels of circulating released antigens, including autoantigens, due to progressive and significant insult, antigen-antibody complexes are formed which gives misleading low titer levels in traditional assays (i.e., ELISA and microarray), since the Ig is already bound in the serum. It may also suggest that these autoantibodies are bound to cellular targets* in vivo*. In our own work, we have noted these decreases followed by rebound in chronic progressive neuropathologies.

It should be noted that in the context of environment-AID and environment-NAb interactions many of these issues remain to be addressed.

## 5. Challenges

### 5.1. Neurotoxicity of Nanoparticles

Linking environmental exposures to ND and AID is a challenge, particularly with the increase in both disorders. Ironically, the development of biomarkers of effects for the vast number of industrial and pharmaceutical chemicals remains a work in progress, yet with technological progress we continue to introduce new materials. The emergence of nanotechnology, which takes advantage of the unique physicochemical properties of submicron-sized nanomaterials, has profoundly impacted every aspect of daily life in the 21st century. In biomedical fields, the demand for nanotechnology and its applications is rapidly growing. Evidence for rapid growth of nanotechnology is reflected by the increase in the annual budget for the National Nanotechnology Initiative from $650 million in 2005 to $1.7 billion in 2014 [27] and by its annual growth rate of more than 17% [28, 29]. The National Science Foundation estimated that in the near future half of all pharmaceutical industry products will have some association with nanotechnology [30]. In spite of public concerns over their potential health impacts [31–35], comparatively little effort has been devoted to understanding the safety profiles of these nanomaterials. From a toxicology perspective, nanoparticles (NPs) possess important characteristic features, which differ from the features of their native parent materials and are greatly influenced by the formulation of NPs. Hence, there is a clear need to better understand the potential adverse health effects associated with emerging nanotechnologies, their biocompatibility, and potential toxicity.

Oberdörster and his collaborators [36–38] and Elder et al. [39] have demonstrated and reviewed studies that inhaled and possibly ingested or topically applied ultrafine particles can be translocated to the brain and nervous system. This translocation is likely to be via the olfactory bulbs and/or the systemic circulation. In the latter case this would involve movement across the blood-brain barrier (BBB), a key strategy for drug delivery using NP [40, 41]. Because of their size, which falls within the same range as viruses, it is possible that systemically distributed NP may also gain access to the nervous system at the neuromuscular junction (NMJ), similar to the poliovirus [42]. Transport whether from the olfactory nerves, the NMJ, or within the brain would likely rely on retrograde axonal transport (RAT) and fast anterograde axonal transport (FAT). Assessment of possible neurodegenerative changes or neurotoxic potential of NP is relatively new. Combustion-derived NPs are capable of being translocated to the brain. One example, manganese oxide, generated during arc welding, may be an occupational contributor to PD in susceptible individuals [43].

**Figure 6:**
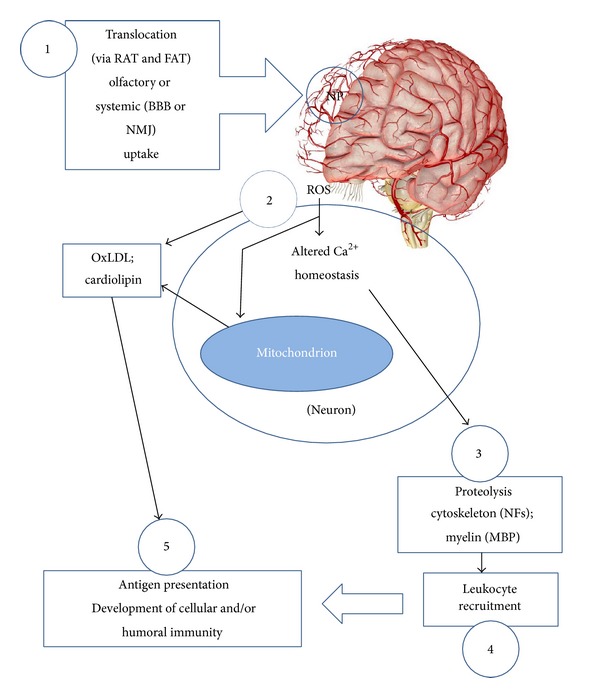
Mechanisms by which NP produce neurotoxicity and resulting autoantibody generation. (1) Translocation of NP introduced via the olfactory bulbs (or neuromuscular junction: NMJ) to the nervous system via retrograde (RAT) and fast anterograde (FAT) transport; (2) translocated NP may induce lipid peroxidation (e.g., LDL) and oxidative stress, as well as Ca^2+^ overload and displacement from ER/mitochondria; (3) cytoskeletal proteolysis, as a result of Ca^2+^ overload, which also results in mitochondrial derangement (e.g., cardiolipin dissociation). These events result in exposure of autoantigens; (4) recruitment of immune effectors, including microglia,* in situ*, and peripheral leukocytes; (5) antigen processing, presentation, and antibody production in lymphoid tissue.

In several reviews and opinion papers [44, 45], it has been suggested that NP may precipitate autoimmune responses in exposed individuals by acting as haptens, exposing cytoskeletal elements, or promoting degeneration as a consequence of calcium overload. Based on published evidence, we have hypothesized that NPs and agents not normally considered neurotoxic may induce NS insult if generated as NPs (Figure 6).

In a preliminary study using arc-spark generated nickel NPs (≤40 nm; Ni-NP), a metal not typically associated with neurotoxicity, mice were assessed for the generation of NAb against NF, GFAP and MBP following chronic inhalation exposure of 6 weeks. The mice used included wild type C57BL/6 and their APO^−/−^ (knockout counterpart). The choice of this model for neurotoxicity studies of NP is to provide a model of a human population susceptible to neurodegeneration. ApoE^−/−^ mice have been shown to be susceptible to excitotoxicity in models of AD [44, 45], hyperphosphorylation of* tau* [46, 47], sensitivity to synaptic derangement [48, 49], and an increased susceptibility to oxidative damage [50]. These are believed to mimic changes associated with aging and increased susceptibility to nervous system damage following trauma and stroke. In addition, although there are apparently no differences in lymphocyte populations, ApoE^−/−^ mice tend to respond stronger to antigen challenge and may be predisposed to autoimmunity [51], including the development of autoantibodies to nervous system antigens [52].

**Figure 7:**
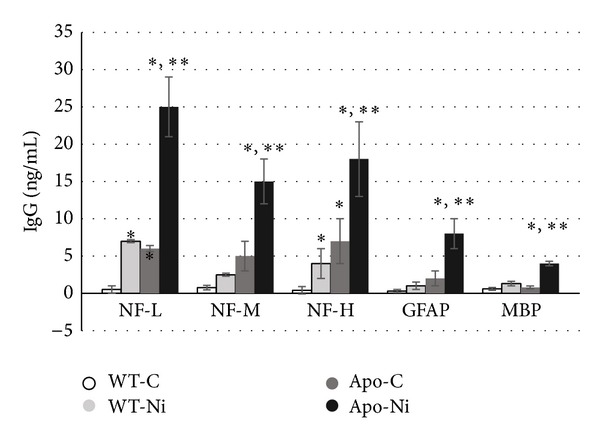
Inhalation exposure of wild-type C57BL/6 (WT) and ApoE knock out male mice to arc-spark generated Ni-NP (≤40 nm; 5 h/wk for 6 wks) resulted in detectable levels of IgG against neurotypic (NF) and gliotypic (GFAP and MBP) proteins. Levels of IgG were significantly (*P* < 0.05) higher in ApoE^−/−^ mice exposed to Ni-NP compared to WT controls, WT exposed to Ni-NP (^∗^) or ApoE^−/−^ control mice (^∗∗^) (inhalation exposures conducted by L. C. Chen and P. Gillespie at New York University Institute of Environmental Medicine).

Titer levels, both IgM and IgG, were detected in sera of mice. Levels of NAb and IgG are shown in Figure 7. While wild type mice exposed to Ni-NP had detectable levels of NAb against all antigens, the more susceptible ApoE null mice had significantly higher titers. In addition titers of anticardiolipin (ACA) and antioxidized LDL (oxLDL) were also detected (not shown).

OxLDL has been shown to accumulate in astrocytes following cerebral infarcts and stimulates IL6 release from astrocytes in culture [53], while ACA have been shown to reduce viability of neuronal cultures [54] and damage to cerebral white and gray matter and inhibit astrocyte function [55, 56].

With the increased development and use of NP in industry and as therapeutic delivery systems, the safety of nanoengineered particles and anthropogenic NP (i.e., ultrafine particle pollutants) should be a priority. Utilizing NAb detection, particularly with inhalation exposure, would provide a cost-effective option in determining neurotoxicity.

### 5.2. Angiogenesis, Neurodegeneration, and Autoimmunity

Angiogenesis, the elaboration of neovasculature, has emerged as playing a central role on both ND and AID. Despite this and the significant role played by vascularization in neurodevelopment, maintenance of the nervous system, and inflammatory autoimmunity, it remains a neglected area of research in the field of toxicology, outside the sphere of carcinogenesis.

Recently completed studies from the Neurotoxicology Laboratory and Pharmaceutical Research Institute have demonstrated that neurotoxic thyromimetic PCB induce angiogenesis through the *α*v*β*3 integrin receptor. Not only is this receptor responsible for mediating proangiogenic activity of thyroid hormone, but it is also targeted by vascular endothelial growth factor (VEGF) and basic fibroblast growth factor (bFGF). The *α*v*β*3 integrin receptor also activates Th17 lymphocytes. The role of these cells in neuroinflammation and neuroimmunity has recently been reviewed by Vojdani et al. and Marwaha et al. [57, 58]. Th17, designated as such because of the production of IL-17, may be major effector of autoimmunity [59, 60], including the production of antibodies, where they provide B help [61, 62]. In addition, recent evidence implicates Th17 and ND such as PD, AD, and MS [63, 64]. Relevant to the toxicity of PCB is evidence that Th17 differentiation and activity may be mediated via *α*v*β*3 integrin receptors in experimental autoimmune encephalopathy [65] or via the aryl-hydrocarbon receptor (AHR) [66], of which several PCB congeners are agonists. In addition, IL-17 has been shown to induce VEGF release, thereby contributing to encephalopathy in SLE [67]. Furthermore, the participation of VEGF and angiogenesis in AID, reviewed by Shoenfeld's group [68], plays a significant role in SLE, RA, and MS. In ND, angiogenesis and hypervascularization mediated by VEGF and *α*v*β*3 integrin receptor-dependent process with ensuing hyperpermeability has been reported [69–77]. In addition, activation of the *α*v*β*3 receptor plays a role in recruitment of leukocytes in response to CCL2 (aka MCP-1) produced from astrocytes [78] and in response to ICAM and VCAM [79]. This is consistent with the localization of *α*v*β*3 integrin to T and B lymphocytes for interaction with the vitronectin matrix during migration and endothelial interactions [80]. The participation of *α*v*β*3 integrin receptor in angiogenesis and immune cell migration and activation in AID underlies its promising potential as a therapeutic target in RA [81] and experimental glomerulonephritis [82].

In the context of the NS, aside from angiogenesis, *α*v*β*3 is implicated in upregulation of glutamate receptor production and excitotoxicity [83, 84], major effector of neurotoxicity and ND [5]. It is relevant that some PCB increase extracellular glutamate availability by inhibiting its uptake into astrocytes [85]. *α*v*β*3 integrin is also implicated in reactive astrogliosis, a hallmark of neurotoxicity, and inhibition of neurite growth and process retraction [86], while *α*v*β*3 integrin receptors play a role in microglia-induced neuroinflammation models of AD, PD, MS, and ALS [87–89].

Taken together, hypervascularization in the nervous system, as demonstrated in the pathogenesis of ND, and increased permeability may provide opportunities for toxicant entry and NAb penetration into the CNS, thereby exacerbating neuropathology.

## 6. Conclusion

Emerging recognition that environmental agents may play a role in ND and AID and that many ND have an autoimmune and/or autoinflammatory component provides not only potential targets of intervention but also biomarkers of effect. Defining these interactions and capitalizing on the humoral immune response, in the form of autoantibodies, provide accessible markers for predicting and diagnosing neurological outcome. As epiphenomena, secondary to initial insult, regardless of etiological factor(s), these NAbs may be indices of neurotoxicity and neurodegenerative processes, as well as a means of monitoring therapeutic efficacy. The potential contribution of NAb to the pathogenesis of ND and neurotoxicity, whether through complement activation, activation of phagocytes, or exacerbation of neuroinflammation, needs to be delineated. This may be a key to understanding disease progression and developing effective interventions in environmentally induced disease. Work over the last two decades in neurotoxicology has concentrated, for the most part, on the use of NAb in preclinical studies and remains to be validated in large cohorts of at-risk humans. In attempts at clinical translation, it should be recognized that immune responses in the presence of exposure may not adhere to the dogma of dose response. It is also important that one should not lose sight of the reality that environmental agents often demonstrate pleiotropic activity, impacting multiple systems. It should also be noted that cellular immune response remains unexplored in the context of neurotoxicology.

In tandem with the use NAb of as potential biomarkers, initial studies have begun to explore the pathogenicity of NAb generated in response to some environmental chemicals. It is also believed that development and validation of biomarkers that capitalize on the immune response may prove useful for determining the safety and/or potential toxicity of emerging technologies (i.e., nanoengineered materials).

Finally, for both the neurotoxicologist and immunotoxicologist, a long neglected area of research at the crossroads of ND and AID is angiogenesis and the effect of chemicals, directly and indirectly, in promoting increased vascular permeability and providing an avenue of disease exacerbation.

## Figures and Tables

**Table 1:** Serum/plasma half-life of some proteins proposed as biomarkers of neurodegeneration.

Protein	Half-life	Reference
Neuron-specific enolase (NSE)	48 hr	[9]
S100*β*	20–120 minutes	[10, 11]
Glial fibrillary acidic protein (GFAP)	10–17 hr	[12]
Myelin basic protein (MBP)	4 hr (plasma) 12 minutes (serum)	[13]

**Table 2:** Protein antigens, their cellular source, and function.

Cell	Protein	Function
Neuron	Neurofilament (NF) Triplet∗	Neuronal intermediate filaments (IF)
	NF-L (light; NF-68)	Mechanical stability of soma, dendrites, and axon
	NF-M (medium; NF-160)	Mechanical stability of soma, dendrites, and axon
	NF-H (heavy; NF-200)	Together with NF-L and NF-M, mechanical stability of the axon

Astrocyte	Glial fibrillary acidic protein (GFAP)	IF of mature astrocytes biomarker of reactive gliosis
	Vimentin	IF transiently expressed during development

Myelinating cells	Myelin basic protein (MBP)	Compaction protein of myelin in CNS and PNS
Oligodendroglia	Myelin oligodendrocyte protein (MOG)	CNS myelin
Schwann Cell	Peripheral myelin protein-22 (PMP-22)	PNS myelin

*Individual proteins differ in their immunogenicity.

**Table 3:** Pearson's correlation coefficients for antineurofilament IgG titers and changes in gait length and width of hens monitored for 21 days^∗^.

IgG	Stride length		Stride width	
	*r*	*P*	*r*	*P*
Anti-NF-L	−0.45	0.04	0.52	0.01
Anti-NF-M	−0.47	0.04	0.48	0.03
Anti-NF-H	−0.33	0.10	0.42	0.05
Anti-GFAP	−0.11	0.63	0.21	0.36
Anti-MBP	−0.23	0.31	0.34	0.13

*Total gait measurements; *n* = 21 measured gaits.
